# Plant Development of Early-Maturing Spring Wheat (*Triticum aestivum* L.) under Inoculation with *Bacillus* sp. *V2026*

**DOI:** 10.3390/plants11141817

**Published:** 2022-07-10

**Authors:** Galina V. Mirskaya, Yuriy V. Khomyakov, Nataliya A. Rushina, Vitaliy E. Vertebny, Elena P. Chizhevskaya, Vladimir K. Chebotar, Yuriy V. Chesnokov, Veronika N. Pishchik

**Affiliations:** 1 Agrophysical Scientific Research Institute, Grazhdansky Pr. 14, 195220 St. Petersburg, Russia; himlabafi@yandex.ru (Y.V.K.); ampollina@yandex.ru (N.A.R.); verteb22@mail.ru (V.E.V.); yuv_chesnokov@agrophys.ru (Y.V.C.); 2 All-Russia Research Institute for Agricultural Microbiology, Podbelskogohwy 3, Pushkin, 196608 St. Petersburg, Russia; chizhevskaya@yandex.ru (E.P.C.); vladchebotar@rambler.ru (V.K.C.)

**Keywords:** PGPB, epiphytic *Bacillus* sp., wheat *Triticum aestivum* L., early maturing, productivity, duration of developmental phases, phytohormones, auxin, gibberellin

## Abstract

The effect of a plant growth-promoting bacterium (PGPB) *Bacillus* sp. *V2026*, a producer of indolyl-3-acetic acid (IAA) and gibberellic acid (GA), on the ontogenesis and productivity of four genotypes of early-maturing spring wheat was studied under controlled conditions. The inoculation of wheat plants with *Bacillus* sp. *V2026* increased the levels of endogenous IAA and GA in wheat of all genotypes and the level of trans-Zeatin in Sonora 64 and Leningradskaya rannyaya cvs but decreased it in AFI177 and AFI91 ultra-early lines. Interactions between the factors “genotype” and “inoculation” were significant for IAA, GA, and trans-Zeatin concentrations in wheat shoots and roots. The inoculation increased the levels of chlorophylls and carotenoids and reduced lipid peroxidation in leaves of all genotypes. The inoculation resulted in a significant increase in grain yield (by 33–62%), a reduction in the time for passing the stages of ontogenesis (by 2–3 days), and an increase in the content of macro- and microelements and protein in the grain. Early-maturing wheat genotypes showed a different response to inoculation with the bacterium *Bacillus* sp. *V2026*. Cv. Leningradskaya rannyaya was most responsive to inoculation with *Bacillus* sp. *V2026*.

## 1. Introduction

Under conditions of rapid population growth and climate change, it is essential to ensure food security by increasing the productivity of strategically important grain crops. Wheat is cultivated in many regions of the world, providing more than 50% of dietary energy needs [1]. For wheat plants, early maturity is one of the major mechanisms of avoiding damage by phytopathogens, the destructive effects of summer droughts and dry hotwinds, late spring and early autumn frosts, and the harm associated with excessive moisture during grain maturing [2,3,4]. The developmental rate of soft wheat is mainly ensured by *VRN* and *PPD* genetic systems, which control the hereditary variability of plant response to vernalization and photoperiod [5,6,7]. *VRN* and *PPD* genes and their combinations affecting growing season duration and heading time could also affect the productivity of common wheat [3,8,9]. A negative correlation between earliness and the number of wheat grains, productive tilling, thousand-grain weight, and the harvest index capacity has been noted [2,10,11,12]. Therefore, increasing the yield of early-maturing wheat cultivars and lines is an urgent problem for agriculture.

A possible way of increasing the productivity of grain crops in sustainable agriculture is the use of biological preparations based on PGPB [13,14,15,16]. PGPBs stimulate plant growth and development through various mechanisms: (1) directly affecting plant growth, e.g., by phytohormone production [17,18,19], ACC deaminase activity, nitrogen-fixing activity [19,20,21,22], and solubilization of potassium, phosphorus, zinc, etc.; and (2) affecting plant growth indirectly through the production of hydrolytic enzymes, HCN, siderophores, and antibiotics [18,23], induced systemic resistance, and biofilm formation [22,24,25]. Up to 80% of bacteria inhabiting the plant root zone (rhizobacteria) synthesize auxins, which stimulate root cell proliferation and increase host plant uptake of minerals and nutrients from the soil [26]. PGPBs can also produce cytokinins, gibberellins, or both for plant growth promotion [17,23,27,28].

Bacteria from the genus *Bacillus* are among the particularly promising PGPBs. Besides the rhizosphere, they can also live on the surface of the aboveground organs of plants and within plant tissues [15,29]. *Bacillus* spp. promote plant growth by producing phytohormones, siderophores, lipopeptides, polysaccharides, and enzymes [17,20,23,25]. They also affect plant homeostasis by regulating the proportion of antioxidant enzymes, both under natural plant growth conditions and under various stresses [21,30,31]. Most studies have reported positive effects of *Bacillus* spp. on wheat growth and productivity [14,32,33,34]. However, the effects of PGPB, and *Bacillus* spp. in particular, on the rate of wheat development are currently poorly understood and require detailed study. The results available in the literature are contradictory, with both the acceleration of developmental phases in different plants under the influence of PGPB [35,36,37,38] and delayed development [39,40] being reported. These controversial results may be due to different plant hormonal changes caused by PGPB since phytohormones play a pivotal role in various developmental processes in plants [41]. The response of early-maturing spring wheat to bacterial inoculation is not clear because there are insufficient results in the literature on the inoculation with PGPB of early spring wheat. A slight stimulation of the root growth of early-maturing wheat cv. Kazakhstanskaya 10, when inoculated with bacteria *Bacillus subtilis 11 BM*, was noted on the 30th day of growing [42].

The application of exogenous GA3 significantly promoted the elongation of the root, stem, and leaf cells [43], enhanced expression of cell elongation genes [44], promoted GA biosynthesis [45], and shortened germination time [46] and the time to flowering [47,48] in various plants, including wheat. We proposed that GA-producing *Bacillus* sp. *V2026* can reduce the duration of developmental phases of early-maturing wheat by increasing plant endogenous GA. Various wheat genotypes have been studied to select the optimal yield genotype/bacterial combination. The optimization of such a combination is important to achieve higher wheat productivity.

The paper presents a study of the effect of *Bacillus* sp. *V2026* bacteria on the hormonal status and the development of different genotypes of early-maturing wheat.

## 2. Results

### 2.1. Bacterial Identification

The bacterium was isolated from the rhizosphere of wheat plants of cv. Leningradskaya 6. The bacterium was a Gram-positive single spore-forming bacillus identified by 16S rRNA and ITS fragment as *Bacillus* sp. *V2026* (the sequences were submitted to the NCBI databases with accession numbers OM764631 and OM855550, respectively). This bacterium is catalase-positive and oxidase-negative; indole and H_2_S are not produced. The Voges–Proskauer reaction is negative. The bacterium can utilize glucose, sucrose, xylose, arabinose, maltose, sorbitol, and mannitol. *Bacillus* sp. *V2026* showed antifungal activity against the phytopathogenic micromycetes *Fusarium oxisporum* and *Fusarium culmorum* (zone with diameters of 30–40 mm). *Bacillus* sp. *V2026* did not mobilize phosphates. The bacterium showed a phytohormonal activity, producing 43.09 ± 0.35 µg/mL IAA and 20.8 ± 0.41 ng/mL GAS3, and did not produce tZ (Table S1).

### 2.2. Identification of Alleles VRN-1 and PPD-D1 Loci

The main loci of the *VRN* and *PPD* genetic systems that determine different degrees of sensitivity to vernalization and photoperiod and, consequently, different rates of development were identified in the plant material using molecular markers specific to the *VRN* and *PPD* genes (Figure S1).

Molecular genetic analysis showed that ultra-early lines AFI177 and AFI91 gave four PCR fragments: 715-bp and 624-bp for *Vrn-A1a* alleles, 1124-bp for the *Vrn-B1a* allele, and 1671-bp for the *Vrn-D1* allele. The cultivars S64 and LR contain PCR products 1671-bp and 1124-bp, respectively, which may correspond to the *Vrn-D1* and *Vrn-B1* alleles. We also found that the tested cultivars S64 and LR contain the *Vrn-A1a* allele since all of them gave 715-bp and 624-bp PCR products with primers *VRN1AF//VRN1-1R*. Cv. S64 yielded a 414-bp PCR fragment, indicating the presence of the photoperiod-insensitive *Ppd-D1a* allele. Cv. LR differed from the other genotypes by the presence of a 288-bp fragment, indicative of the photoperiod-sensitive *PPD-D1b* allele (Figure S1).

### 2.3. Yield and Yield Components

The effects of inoculation of the *Bacillus* sp. *V2026* on the productivity components of wheat plants are presented in Table 1.

The results suggest that yield increase after bacterial inoculation was mainly due to an increase in productive tilling capacity: the maximum value was observed in early-maturing cultivars S64 and LR, by 24.6% and 27.3%, respectively (Table 1). Plants of cv. LR characterized by the highest tilling capacity were the most responsive to inoculation judging by this characteristic. The proportion of productive tilling increased in inoculated plants of lines AFI177 and AFI91 by 21.1% and 15.0%, respectively. The effect of inoculation with *Bacillus* sp. *V2026* on plant height was observed only in the AFI91 line; the height increased by 5.8%, but no significant effect on the height of S64, LR, and AFI177 plants was noted.

Spike length significantly increased in inoculated S64 and LR plants: by 9.0% and 6.3%, respectively. Moreover, plants of these cultivars had a longer spike in the control variant. Plants of AFI91 and AFI177, which had a shorter spike, had no significant effect of *Bacillus* sp. *V2026* on the length of the spike.

The number of spikelets in the spike statistically increased significantly in inoculated plants of all genotypes, from 5.6% in AFI91 to 7.5% in LR. Inoculation with *Bacillus* sp. *V2026* was strongly influenced by increasing the number of grains of the spike in early-maturing cultivars S64 and LR (by 21.0% and 16.7%, respectively) than in ultra-early-maturing lines AFI91 and AFI177 (by 8.8% and 8.9%, respectively). The responsiveness of AFI91 and AFI177 plants to inoculation with *Bacillus* sp. *V2026* by grain weight of the spike (22.3% and 27.0%, respectively) was greater than S64 and LR plants (17.2% and 13.5%, respectively).

**Figure 1:**
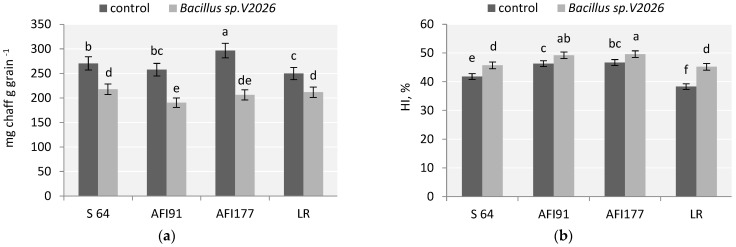
Effect of inoculation with *Bacillus* sp. *V2026* on chaff to grain ratio (**a**) and harvest index (HI) (**b**). The bars are means of two experiments with 50 biological replications per variant. Bars show ± SEM, and different letters (a–f) show a significant difference at the *p* ≤ 0.05 level, as determined by Duncan’s multiple test.

A stimulating effect of *Bacillus* sp. *V2026* on the number of tillers and spike productivity contributed to a higher number of grains per plant (from 16.6% in API177 and up to 57.8% in LR) and grain weight per plant (from 32.5% in S64 and up to 62.2%, in LR) in all early-maturing genotypes. Inoculation with *Bacillus* sp. *V2026* resulted in a greater decrease in the ratio of chaff in the spike of AFI91 and AFI177 plants (by 26.2% and 30.5%, respectively) than in the spike of S64 (19.5%) and LR (15.2%) plants (Figure 1a).

An increase in wheat grain yield after inoculation with *Bacillus* sp. *V2026* was accompanied by an increase in total biomass (Table 1). Ultra-early-maturing lines AFI91 and AFI177 were the most responsive to bacterial treatment in terms of total dry weight of plants and straw yield, the latter increasing by 27.1% and 26.5%, respectively. HI (harvest index) in the control varied from 38.3% in LR to 47.7% in AFI177. Inoculation with *Bacillus* sp. *V2026* led to a statistically significant increase in HI in all studied cultivars and wheat lines, from 4.2% in AFI177 plants to 18.4% in LR plants (Figure 1b). Two-way ANOVA analysis revealed that all wheat yield components examined in our study were statistically significantly influenced (*p* ≤ 0.05) by both genotypic differences and inoculation with *Bacillus* sp. *V2026*, whereas the interaction of these factors influenced only grain number per spike, number and weight of grains per plant, and HI (Table S2).

### 2.4. Protein and Macronutrient/Micronutrient Content in Wheat Grain

Genotype-related variation in the macro- and microelement content of grain of early-maturing wheat genotypes investigated in our study was relatively low (Table 2).

Analysis of the experimental data showed that inoculation with *Bacillus* sp. *V2026* had a strong influence on the accumulation of mineral macronutrients (N, P, K) and micronutrients (Fe, Mg, Zn, and Mn) in wheat grain (Table 2). Significant differences between the genotypes were found for the majority of analyzed mineral elements involved in the analysis. The results suggest that bacterial treatment increases N content from 6.2% in cv. LR to 20.1% in line AFI177 and in K content from 6.9% in cv. LR to 22.9% in cv. S64. Statistically significant differences in the concentration of P in the grain were found in plants of ultra-early lines AFI91 (10.7%) and AFI177 (9.7%), while in plants of cv. S64 and cv. LR, this increase remained insignificant. AFI91 and AFI177 lines were also the most responsive to inoculation with *Bacillus* sp. *V2026* in respect of the content of Fe, Mg, Zn, and Mn in grain. In cv. S64, inoculation resulted in significant changes in Mn concentration (7.2%) but did not have a strong influence on Fe, Mg, and Zn concentrations. Cv. LR was the least sensitive to *Bacillus* sp. *V2026* in terms of changes in concentration of microelements in the grain.

**Figure 2:**
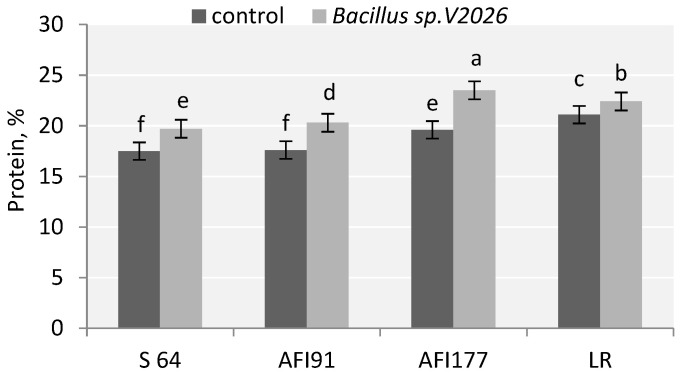
Effect of inoculation with *Bacillus* sp. *V2026* on content of grain protein of early-maturing wheat. Bars with different letters are significantly different at *p* ≤ 0.05, as determined by Duncan’s multiple range test.

Inoculation with *Bacillus* sp. *V2026* had an effect on grain quality: the protein content in the grain increased significantly in all early-maturing genotypes. These changes were more discovered in lines AFI177 (19.9%) and AFI91 (15.3%) (Figure 2). At the same time, LR and AFI177 genotypes were characterized by a higher grain protein content than S64 and AFI91 genotypes, both in the control variant and in the variant with treatment.

### 2.5. Duration of Developmental Phases

Our results showed that the difference in the heading time between ultra-early lines AFI177 and AFI91 and early-maturing cultivars S64 and LR made up 5–9 days (Table 3). Plants of cv. S64 and cv. LR had a longer “stem elongation–heading” period and a shorter “heading–maturing” period compared with plants of the AFI91 and AFI177 lines.

We also assessed the effects of inoculation with *Bacillus* sp. *2026* on the pattern of development of early-maturing genotypes (Table 3). Inoculation with the bacterium significantly shortened the time to reach each growth stage in early-maturing genotypes. Maturation of inoculated plants occurred on average 2–3 days earlier than in control plants, the difference being significant. The number of days from seedling to each growth stage was less in the API91 line than in other genotypes. It is also noteworthy that cv. LR was the most responsive to inoculation with *Bacillus* sp. 2026, showing the largest reduced duration of the period from seedling to maturing. Heading of inoculated plants of cv. S64, API177, API91, and cv. LR occurred earlier, by 1.5, 2.4, 1.4, and 3.1 days, respectively, than in plants without bacterial treatment, with the cycle reduction being greater from seedlings to stem elongation. Cv. S64, API91, and cv. LR were not responsive to *Bacillus* sp. *V2026* inoculation at the stage of “stem elongation–heading.” The duration of the period from heading to maturing in all four genotypes practically did not differ in the control and experimental variants.

Two-way ANOVA analysis showed that the factor “genotype” had a significant impact on the duration of all studied periods of wheat ontogenesis. At the same time, only genotypic differences contributed significantly to variability in the periods “stem elongation–heading” and “heading–maturing.” The factor “*Bacillus* sp. *V2026*” significantly influenced the duration of the initial stages of ontogenesis from seedlings to tillering and from tillering to stem elongation, as well as the timing of heading and maturing (Table S2). The interaction of the factors had a significant effect on the duration of the seedlings–stem elongation and seedlings–heading periods.

### 2.6. Seedling Growth

Inoculation with *Bacillus* sp. *V2026* increased root and shoot lengths in 14-day-old wheat seedlings of early-maturing genotypes (Figure S2). The relative increase in root length in LR, S64, AFI91, and AFI177 seedlings due to bacterial inoculation made up 20.7%, 11.4%, 20.4%, and 19.1%, respectively, compared with the control (Table 4). The root biomass in these variants increased from 11.0% (S64) to 23.0% (AFI177). AFI177 and LR were more responsive to inoculation. The responsiveness of shoots to the action of the bacterium was less pronounced. The shoot length of cv. LR and cv. S64 treated with *Bacillus* sp. *V2026* was 10.3% and 5.9%, respectively, greater than in control plants. Inoculated plants of AFI177 and AFI91 lines had no statistically significant differences in shoot length compared with the controls (Table 4). A significant increase in shoot biomass after inoculation was observed in AFI177, LR, and S64 seedlings: by 18.8%, 13.4%, and 8.7%, respectively. Thus, inoculation with *Bacillus* sp. *V2026* stimulated the accumulation of root biomass more considerably than the accumulation of shoot biomass. The root/shoot weight ratio increased from 2.9% (S64) to 7.5% (LR).

The wheat genotypes involved in our study responded to inoculation with *Bacillus* sp. *V2026* in a different manner. Cv. LR and line AFI177 were more sensitive to inoculation with this strain. Inoculation stimulated roots and shoots of LR seedlings, shoots of AFI177 seedlings, and roots of AFI91 seedlings. Cv. S64 was the least responsive to bacterial inoculation compared with other cultivars and lines.

### 2.7. Photosynthetic Pigments and Lipid Peroxidation

**Figure 3:**
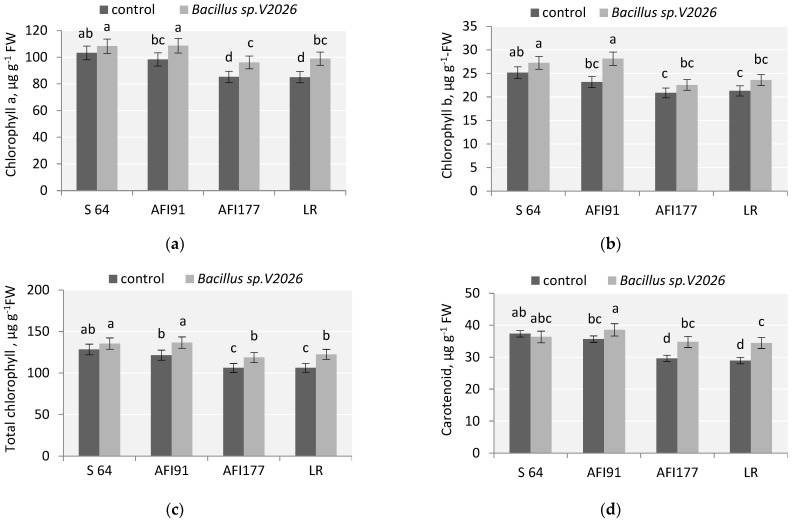
Effect of *Bacillus* sp. *V2026* on chlorophyll a (**a**), chlorophyll b (**b**), total chlorophyll (**c**), and carotenoid (**d**) content of early-maturing wheat plants grown under hydroponic conditions. Bars with different letters are significantly different at  *p* ≤ 0.05, as determined by Duncan’s multiple range test.

**Figure 4:**
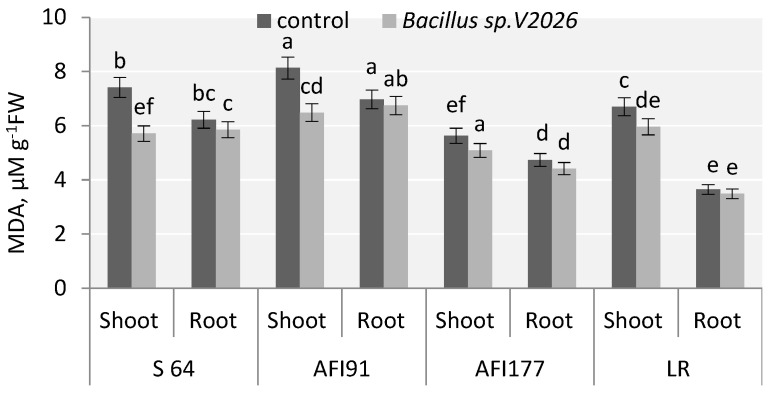
Effect of *Bacillus* sp. *V2026* on malondialdehyde (MDA) content of early-maturing wheat plants grown under hydroponic conditions. Bars with different letters are significantly different at *p* ≤ 0.05, as determined by Duncan’s multiple range test.

The physiological state of plants after inoculation with *Bacillus* sp. *V2026* was determined by measuring the chlorophyll content and the level of malonic dialdehyde (MDA). Data on the effect of *Bacillus* sp. *V2026* on various biochemical parameters are shown in Figure 3 and Figure 4. The concentration of chlorophyll and carotenoids in leaves significantly increased after bacterial treatment (Figure 3). The increase in total chlorophyll (Chl) and chlorophyll a (Chl a) in samples of AFI91, AFI177, and LR made up, respectively: 12.5%; 11.8%, 15.2% and 10.5%, 12.7%, 16.4% (Figure 3). A statistically significant increase in chlorophyll b (Chl b) levels (21.1%) was observed only in inoculated seedlings of the ultra-early-maturing line AFI91. No significant changes in the content of total Chl, Chl a, and Chl b in the leaves of inoculated S64 plants were noted. An increase in carotenoid content (Car) was observed in LR, AFI177, and AFI91 plants, by 19.4%, 17.6%, and 8.1%, respectively, as compared with the controls. In S64 plants, the level of Car tended to decrease after inoculation. The highest average content of total chlorophyll was registered in cv. S64 and line AFI91 in both the control and the experimental variants.

One of the metabolites of lipid peroxidation is malondialdehyde (MDA); a statistically significant decrease in MDA level in the shoots of inoculated plants was registered. The decrease was the most obvious in S64 and AFI91 plants: by 22.9% and 20.3%, respectively (Figure 4). Inoculation with *Bacillus* sp. *V2026* tended to decrease MDA levels in the roots of all genotypes involved in the study, ranging from 3.3% in AFI91 to 6.8% in AFI177.

### 2.8. Endogenous Levels of Plant Hormones in Seedlings

**Figure 5:**
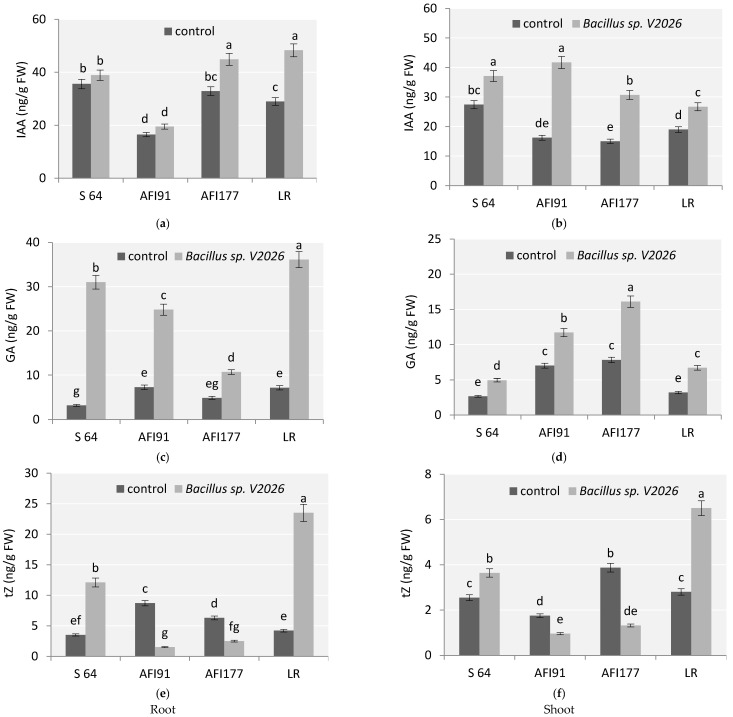
Effect of *Bacillus* sp. *V2026* on concentrations of plant hormones: (**a**,**b**) indole-3-acetic acid (IAA); (**c**,**d**) gibberellic acid (GA); and (**e**,**f**) trans-Zeatin (tZ) in roots (**a**,**c**,**e**) and shoots (**b**,**d**,**f**) of early-maturing wheat plants grown under hydroponic conditions. Bars with different letters are significantly different at *p* ≤ 0.05, as determined by Duncan’s multiple range test.

This study revealed that with inoculation *Bacillus* sp. *V2026*, the content of endogenous hormones in wheat plants significantly varied depending on the genotype (Figure 5). An increase in indolyl-3-acetic acid (IAA) and gibberellic acid (GA) content was observed for all genotypes studied. The most evident change in IAA levels was noted in the roots of cv. LR (by 66%) (Figure 5a) and shoots of lines AFI177 and AFI91 (2–2.5-fold, respectively) (Figure 5b). A high level of basal auxins in shoots was detected in cv. S64 (Figure 5b). Plants of line AFI91 differed significantly from other genotypes in having a reduced basal level of IAA in the roots (Figure 5a).

The most significant increase in GA was observed in the roots of plants of early-maturing cultivars LR and S64 (Figure 5c). The basal level of GA in the roots differed significantly between cv. S64 and cv. LR, as well as between cv. S64 and line AFI91. The basal level of GA in shoots of lines AFI177 and AFI91 was higher than in shoots of cv. S64 and cv. LR (Figure 5d).

The content of cytokinins (tZ, trans-Zeatin) also showed significant differences in all four early-maturing wheat genotypes after inoculation compared with the control (Figure 5e,f). Inoculation with *Bacillus* sp. *V2026* significantly increased tZ content in roots and leaves of cv. S64 and cv. LR, whereas in roots and shoots of ultra-early-maturing lines AFI177 and AFI91, a 2.5–5-fold decrease in tZ concentration, respectively, was observed. Changes in tZ content were most noticeable in cv. LR; its level increased two-fold in shoots and five-fold in roots (Figure 5e,f).

Two-way ANOVA revealed that a statistically significant contribution to the variation in the concentration of all endogenous hormones was made by genotype-related differences (factor 1), by inoculation with *Bacillus* sp. *V2026* (factor 2), and by the combination of these two factors (Table S2).

## 3. Discussion

Bacterium *Bacillus* sp. *V2026* was found to produce plant hormones, such as IAA (at a concentration of 40.3 µg·mL^−1^) and GA3 (at a concentration of 20.8 ng·mL^−1^). There are data in the literature on the production of phytohormones IAA (at concentrations 0.1 to 92 µg·mL^−1^) [49,50] and different GA (at concentrations 0.13–17.9 ng mL^−1^) [24] by bacteria from genera *Bacillus*. Thus, concentrations of GA3 57 and 51 ng mL^−1^ were revealed for *B. licheniformis* and *B.pumilus* accordingly [51].

The four early-maturing genotypes of spring wheat studied in this work differed not only in morphological characteristics, yield structure indicators, and duration of individual development phases but also in the combination of allelic forms of the *VRN-1* and *PPD-D1* genes (Figure S1). It is known that different alleles of *VRN-1* and *PPD-D1* and their combinations have different effects on the timing of heading, duration of individual development phases, and yield structure in wheat [3,8,9]. Early-maturing genotypes are characterized by a certain allelic composition of these genes [3,52]. The observed stimulating effect of *Bacillus* sp. *V2026* on grain yield and yield-related traits of early-maturing wheat genotypes is consistent with the results of several other studies on the effect of PGPB on wheat productivity under normal and stress conditions [22,53,54,55,56]. PGPB inoculation has been shown to positively affect grain yield [14,32,54,57,58,59,60], number of tillers, plant height and biomass [14,22,32,34], spike length [53,61], number of spikelets and grains in the spike [54,62,63], weight of thousand grains [57,58,61,63] and the content of nutrients [32,34,57,59,60].

We found that inoculation with *Bacillus* sp. *V2026* and genotypic differences had a statistically significant impact (*p* ≤ 0.05) on all the indices of wheat productivity recorded in our experiments. The interaction of factors had a significant effect on the number of grains per spike, the number and weight of grains per plant, and HI (Table S3). It is of interest that the increase in grain yield of wheat genotypes after inoculation was mainly associated with a higher number of productive shoots and the number and weight of grains per plant. At the same time, the effect on plant height, spike length, and weight of thousand grains was much less significant.

Under the conditions of inoculation with *Bacillus* sp. *V2026*, the main impact to change in the grain yield of early-maturing genotypes was made by the increase in productive tilling capacity (Table 1), which is an important trait determining the yield of wheat. The positive effect of PGPB on shoot formation has been reported in other studies [32,53,54,57,58,61,63]. Cytokinins are central to the regulation of wheat shoot growth, and the IAA/CK ratio is used to determine plant response in this respect [64,65]. Stimulation of productive tilling capacity observed after inoculation with PGPB is associated with their ability to produce IAA and CK, as well as with their influence on the hormonal balance of plants [66]. The evidence suggests that the promotion of productive tilling capacity induced by the *Bacillus* sp. *V2026* could be mediated by bacterial IAA and GAs. Effective stimulation of tiller formation by *Bacillus* sp. *V2026* seems to be associated with its IAA-producing activity, which resulted in an increase in endogenous IAA and tZ in wheat roots and shoots (Figure 5). Tiller numbers increased after inoculation in all the genotypes involved in our study, but the response varied depending on the degree of hormonal changes. The highest stimulation of productive tilling capacity was registered in plants of cv. LR and was associated with more pronounced changes in hormonal levels in roots and shoots of plants of this cultivar, especially changes in the level of CK.

Similar to IAA, CK are among the main regulators of primary root growth due to their participation in cell division and differentiation in the root meristem. Accumulation of CK in inoculated wheat plants has been shown to be associated with an increase in shoot weight [17]. Even though *Bacillus* sp. *V2026* was found to not produce trans-Zeatin, the concentration of cytokinins in shoot and root tissues of plants of early-maturing genotypes after inoculation with the bacterium changed considerably more than IAA concentration. Interestingly, the accumulation of root tZ found in our experiments in cv. S64 and cv. LR did not inhibit root growth (Table 4), although high concentrations of CK are known to do so [67]. However, both the increase in the levels of tZ in the roots of S64 and LR plants and their decrease in roots of AFI177 and AFI91 plants were accompanied by stimulation rather than inhibition of root growth. These ambiguous results can be explained by the literature data indicating that, on the one hand, auxins decrease the levels of CK by inhibiting their synthesis [68], and, on the other hand, the auxin-induced increase in the volume of the root where CK are synthesized promotes the accumulation of cytokinins [62]. Zeatin levels in leaves increased after the inoculation of *A. thaliana* plants with the PGPB strain *Burkholderia phytofirmans*, which has also not been shown to be able to synthesize CK [69]. The increased level of endogenous CK in wheat roots after inoculation with *Paenibacillus illinoisensis* IB 1087 has been explained by its IAA activity [18].

An increase in the productivity of an individual spike associated with an increase in the number and weight of grains is thought to be a promising direction for increasing the productivity of early-maturing genotypes [2]. Grain number is more variable than grain weight [70,71], and the yield is much more often associated with the number of grains than with the average grain weight [72,73]. We found that inoculation with *Bacillus* sp. *V2026* had a greater effect on increasing the number of grains in the spike in S64 and LR, while in lines API91 and API177, it had a greater effect on increasing the grain weight of the spike (Table 2). Grain number in wheat is largely determined in the stem elongation phase, with the process of spike growth before heading being crucial for the number of grains [74,75]. It should be noted that in our study, the duration of the period from stem elongation to heading, which has a decisive influence on spike productivity after inoculation of the plants with *Bacillus* sp. *V2026*, did not change significantly.

The stimulating effect of *Bacillus* sp. *V2026* on stem and spike productivity resulted in a significant increase in the number of grains per plant and plant grain weight in all early-maturing genotypes. A significant increase in grain yield after inoculation with *Bacillus* sp. *V2026* in early-maturing cultivars S64 and LR was determined by more intensive stem formation. In contrast, in API91 and API177 lines, the increase in grain weight of the spike (Table 1) was due to a more effective redistribution of nutrients between the structural components of the spike, as evidenced by a significantly improved ratio of grain/chaff in the spike of plants of these two lines (Figure 1B). It is currently thought that fruiting efficiency and the ratio between the productive and the vegetative components of the spike (which is associated with fruiting efficiency) are among the characteristics important for achieving an increased yield in wheat [12,76,77]. PGPB *Bacillus* sp. *V2026* increased grain weight by intensifying the redistribution of substances between the structural components of the spike. Differences between wheat genotypes in respect of this characteristic were observed.

Inoculation with *Bacillus* sp. *V2026* resulted in a statistically significant increase of HI in all wheat cultivars and lines involved in our study. This increase seems to be associated with a change in donor-acceptor relationships between the spike and the vegetative mass and the redirection of the supply of nutrients mainly towards the spike. We showed that bacteria *Bacillus* sp. *V2026* stimulated nutrient supply to the grain and affected the level of N, P, K, Fe, Mg, Zn, and Mn in wheat grain (Table 2), which is consistent with our earlier results obtained on *Bacillus subtilis N2* [78,79]. Our results accord with those of [14], who reported an increase in the content of macro- and micronutrients in wheat grain after inoculation with *Bacillus megaterium*. There is evidence of positive effects of PGPB on nutrient uptake (mainly N), yield and grain quality [32,34], protein, P, K and Fe concentration in wheat grain [57,59,60].One of the core processes in primary plant metabolism that is directly related to productivity is photosynthesis. The content of Chl and Car, while indirect, is the most important biochemical indicator of plant photosynthetic activity. Our results also showed an increase in Chl and Car content in all wheat genotypes involved in the study (Figure 3) and a decrease in MDA accumulation and, thus, lipid peroxidation, indicating a lowered stress load (Figure 4). Increased concentration of chlorophyll in leaves activates photosynthesis, ensuring a more rapid accumulation of plant biomass. It has been shown that bacterial inoculation can positively affect the content of photosynthetic pigments in plants [61]. This is reflected in the activity of photosynthetic apparatus, which affects the rate of accumulation of assimilates, plant growth, and productivity. Increased chlorophyll content may be associated with positive effects of PGPB on water and mineral uptake [61,80].

It was found that the bacterium *Bacillus* sp. *V2026* had a statistically significant effect on the duration of the vegetation period of ultra-early-maturing lines and early-maturing cultivars of common wheat, resulting in the acceleration of heading and maturating (Table 3).

Inoculation with *Bacillus* sp. *V2026* accelerated the growth of wheat plants by shortening the vegetation period at the early stages of development from seedlings to stem elongation. It did not affect the rate of development at later ontogenetic stages. Bacterium *Bacillus* sp. *V2026* shortened the growing season of early-maturing wheat plants, conceivably due to the production of the GA hormone. It is known that the application of exogenous GA accelerated flowering in winter wheat cultivars [81], significantly influenced spike development, and shortened the duration of the preheading phase in spring cultivars [82,83]. GAs are a major class of phytohormones regulating plant development, from seed germination and vegetative growth (including initiation and stimulation of flowering) to fruit and seed setting [84,85].

In our study, inoculation with *Bacillus* sp. *V2026* impacted the change in endogenous GA, significantly increasing its concentration in roots and shoots of all wheat genotypes involved in the study, with the most significant increase observed in roots (Figure 5). In addition, increased GA concentration was found in plants treated with GA-producing bacteria, confirming their effects on plant hormonal status [23,28]. To note, under conditions of bacterial inoculation, the ultra-early-maturing lines AFI177 and AFI91 demonstrated a higher level of endogenous GA in shoots, while early-maturing cultivars S64 and LR showed a higher level of GA in roots. These differences may be associated both with a more intense growth of ultra-early-maturing genotypes at the early ontogenetic stages (Table 3) and with greater responsiveness of early-maturing cultivars, especially LR, to inoculation (Figure 5).

Similar results were obtained by [38], who showed that inoculation with *Bacillus subtilis* B26 resulted in a shortening of the growing season of *Brachypodium distachyon*. Some PGPBs are known to synthesize gibberellins [23,86], which promotes plant growth. The positive effect of a PGPB *Bacillus methylotrophicus* on plants through the secretion of several gibberellins has been confirmed by the increased percentage of seed germination in lettuce, melon, soybean, and vegetable mustard [87]. GA-producing *Bacillus* sp. strains have been reported to stimulate the growth of red pepper [88] and rice [28].

As shown in our experiments, inoculation of wheat plants with *Bacillus* sp. *V2026*, contributed to a change in the level of endogenous IAA in plant shoot and root tissues (Figure 5). Since plants are capable of auxin uptake from the nutrient medium [18,89], increased concentration of auxins in plants treated with auxin-producing *Bacillus* sp. *V2026* may be attributed to the uptake of microbial hormones by plants and appeared to depend on the characteristics of each genotype. IAA is a phytohormone so important for plant development and growth, performing multiple functions, including the response of roots and shoots to light and gravity [90,91], initiation of lateral and adventitious roots [92,93,94], stimulation of cell division and elongation of stems and roots [95,96], vascular tissue differentiation [96], apical dominance, and flower morphogenesis [97,98]. Auxin levels in lines AFI177 and AFI91 increased to a greater extent in the shoots; those in cv. LR increased to a greater extent in the roots, while the changes in auxin levels in cv. S64 were less pronounced (Figure 5). A weak positive correlation between the increase in IAA concentration and the acceleration of development was observed, indicating a possible effect of bacterial IAA on the ontogenesis duration.

Stimulation of rhizogenesis is one of the best-known effects of auxins [92,99]. The increase in length and weight of the roots observed after inoculation with *Bacillus* sp. *V2026* in our experiments (Table 4) was associated with changes in auxin levels in the roots of plants of all the genotypes involved in the study (Figure 5). A greater increase in root length and weight in LR and AFI177 plants was apparently due to a higher and more stable increase in endogenous IAA in plants of these genotypes after inoculation with *Bacillus* sp. *V2026*. Similar data have been obtained in experiments with inoculation of wheat plants with auxin-producing *Paenibacillus illinoisensis IB 1087* and *Pseudomonas extremaustralis IB-K13-1A*: increased root weight and increased auxin levels in the roots have been registered [18]. Inoculation with IAA-producing *Bacillus* strains on plant roots enhances root length as well as the number of lateral roots [50,53,93]. IAA-producing bacterium *Bacillus* spp. controls endogenous IAA levels in plant roots by regulating auxin-responsive genes, which changes the root architecture [100]. Auxin-producing bacteria are known to enhance both root and shoot growth [101]. Similarly, in our study, inoculation with *Bacillus* sp. *V2026* resulted in an increase in shoot length and weight, with plants of LR and AFI177 genotypes being more susceptible to bacterial inoculation (Table 4). In our experiments, inoculation with *Bacillus* sp. *V2026* resulted in a greater increase in the biomass of roots compared with that of shoots, which contributed to an increase in the root/shoot weight ratio. According to the available literature data, after inoculation of plants with cytokinin (CK)-producing bacteria, an opposite pattern is observed: a greater increase in the biomass of shoots compared with that of roots results in a decreased root/shoot weight ratio [102].

It is important to note that the effect of auxins on shoot growth is seldom discussed, probably due to the fact that IAA transport from roots to shoots is less studied than cytokinin transport. IAA- and CK-producing PGPBs are known to reconstruct the architecture of the root system by altering the hormonal balance of plants [17,25,103,104].

Thus, *Bacillus* sp. *V2026* promotes growth, accelerates development, and increases the yield of early-maturing genotypes of spring soft wheat. The effect of this strain on the duration of wheat ontogenesis is particularly interesting, as it provides an additional opportunity of simulating the timing of heading and maturation of cultivars depending on the region of cultivation. Our results suggest that PGPB *Bacillus* sp. *V2026* stimulate the ontogenesis of early-maturing genotypes by increasing the concentration of endogenous GA in the early stages of wheat ontogenesis. According to data available in the literature, an increase in bioactive GA results in an upregulation of the expression of transcription factors required to initiate the transition of the wheat apical meristem to generative development [105,106]. In general, the findings allowed us to assume that GA activity of *Bacillus* sp. *V2026* explains their ability to influence the GA-dependent signaling pathway, which regulates various aspects of plant development, including the duration of early stages of ontogenesis [82,107,108].

## 4. Materials and Methods

### 4.1. Plant Material

Seeds of early-maturing soft spring wheat (*Triticum aestivum* L.) cv. Sonora 64 (S64) (k-45398) and cv. Leningradskaya rannyaya (LR) (k-142751) were provided for research by the Department of Wheat Genetic Resources of the N.I. Vavilov All-Russian Institute of Plant Genetic Resources (VIR) (St. Petersburg, Russia). Ultra-early-maturing lines of soft spring wheat AFI91 and AFI177 were obtained from the Agrophysical Research Institute [109]. Heading time in these lines is comparable with that of typical representatives of ultra-early-maturing wheat from the VIR collection [2].

### 4.2. Identification of Alleles VRN-1 and PPD-D1 Loci

Using allele-specific primers, the presence of alleles of the *VRN-1* and *PPD-D1* loci in cultivars S64 and LR and lines AFI177 and AFI91 was determined. For molecular genetic analysis, genomic DNA was isolated from 5-day-old seedlings by the CTAB method after [110]. Published allele-specific primers were used to detect dominant and recessive alleles of *Ppd-D1*, *Vrn-A1*, *Vrn-B1*, and *Vrn-D1* genes (Table S3). Reaction mixture preparation protocols and PCR conditions followed the recommendations of the authors of the molecular markers [5,111,112].

### 4.3. Screening and Isolation of PGPB

PGPB were isolated from roots of spring wheat cv. Leningradskaya 6. Flasks containing 100 mL of sterile phosphate buffer and 10 g of wheat roots were placed in an ultrasonic bath (Bandelin; 50 Hz) for 10 min. The phosphate buffer solution containing microorganisms washed from the roots was serially diluted. Then, 0.1 mL of various dilutions was inoculated on Petri dishes containing LB (Luria Bertani, Sigma-Aldrich, St. Louis, MO, USA) agar medium.

### 4.4. Identification and Characteristics of Bacteria

Genomic DNA of *Bacillus* sp. *V2026* was isolated using the Monarch^®^ Genomic DNA Purification Kit (NEB, Ipswich, MA, USA) according to the manufacturer’s protocol.

PCR amplification of DNA fragments was performed according to a standard protocol using universal primers [113,114] (Table S3). PCR parameters were: (1) matrix pre-denaturation—95 °C, 3 min; (2) 30 cycles: denaturation—94 °C, 30 s; primer annealing—54 °C, 30 s; elongation—72 °C, 30 s; and (3) final elongation—72 °C, 5 min. The obtained PCR fragments were isolated from agarose gel [115] and sequenced using an ABI PRISM 3500xl automatic sequencer (Applied Biosystems, Waltham, MA, USA) according to the manufacturer’s protocol. The strain was identified by comparing the obtained nucleotide sequences of the 16S rRNA gene and ITS fragment with the RDP (https://rdp.cme.msu.edu, accessed on 1 June 2022) and GenBank databases (https://blast.ncbi.nlm.nih.gov, accessed on 1 June 2022). The sequences were submitted to the NCBI databases with accession numbers OM764631 and OM855550, respectively.

The Gram reaction was determined using the Gram-staining method with the help of a bioMe’rieux Gram-staining kit. Catalase activity was examined via the production of oxygen bubbles using H_2_O_2_ (3%, *v*/*v*), and oxidase activity was detected using a commercial oxidase strip (Sigma-Aldrich, St. Louis, USA). H_2_S production was determined according to [116]; indole production was assessed by the Ehrlich method [117]. Phosphate solubilizing activity was assayed on Pikovskaya medium [118]. Bacterial biochemical characteristics such as the utilization of D-glucose D-sucrose, maltose, arabinose, D-galactose, xylose, inositol, dulcitol, sorbitol, glycerol, and mannitol were determined according to [119]. For the agar well diffusion assay, PDA (potato dextrose) agar plates containing 10^4^ Fusarium conidia per mL agar were prepared. Then the wells with a diameter of 5 mm were cut in agar. Liquid culture of *Bacillus* sp. *V2026* was added to the wells [120]. The plates were incubated at 28 °C for 72 h and verified every 12 h. The diameter of the zone of inhibition of the growth of the mycelium of the fungus was measured in mm.

The bacterial phytohormones IAA, tZ, and GA in the extract were determined using a VARIAN 212 LC high-performance liquid chromatograph with a mass selective detector (Varian 500 MS system). Detection of IAA was carried out using ESI- (electrospray) ion at 174 *m*/*z*. The detection of tZ and GA3 was carried out using ESI+ for ions at 220 *m*/*z* and 345 *m*/*z*, respectively. To determine phytohormones, 50 mL of liquid culture (and 50 mL sterile liquid medium, used as a control) was taken and centrifuged at a speed of 3000–5000 rpm for 5 min. The supernatant was drained into a dividing funnel. The precipitate was shaken twice with 30 mL of distilled water and centrifuged after combining the supernatant in a dividing funnel. The combined supernatant in the dividing funnel was acidified with a 10% solution of acetic acid to a pH of 2, after which phytohormones were extracted three times with 10 mL of ethyl acetate. The upper ethyl acetate layer was drained through anhydrous sodium sulfate and evaporated until dry on a rotary evaporator at a temperature of no more than 40 °C. The extraction was performed three times. Chromatography was carried out in the gradient mode (phase A, methanol + 0.1% formic acid; phase B, deionized water +0.1% formic acid). The chromatographic system used a Cosmosil C18 4.6 ID 150 mm column. The chromatograph was calibrated using the Sigma-Aldrich internal standards for pure hormone substances. The identification of hormones was carried out in the mass–mass mode.

### 4.5. Experimental Design

#### 4.5.1. Pot Experiments

The plants were grown in vegetative light units with the following parameters: illumination with lamps DNaZ-400 (Reflax, Moscow, Russia), illumination intensity 23–25 klx, 16-h photoperiod, temperature 23–24 °C (day) and 19–20 °C (night), and humidity 70–80%.

The plants were grown in 4 L pots, five plants per pot. The pots were placed randomly in five replications per variant. The experimental design included two variants: wheat plants without treatment (control) and wheat plants treated with *Bacillus* sp. *V2026* (treatment). The experiment was repeated twice. Wheat seeds, after surface sterilization in 70% ethanol for 2 min, were washed with water and placed in Petri dishes for germination in a thermostat at 26 °C for 48 h. Germinated seeds were planted in pots, 10 plants per pot; 7 days after sowing, 5 identical plants per pot were left. Soddy-podzolic light loamy soil was used in the root layer containing mobile phosphorus 198 mg/kg, mobile potassium 112 mg/kg, nitrate nitrogen 18.2 mg/kg, and ammonium nitrogen 34.6 mg/kg.

The PGPB were grown at 28 °C for 48 h at 140 rpm in a broth of Luria–Bertani (LB) medium in a rotatory shaker. The bacterial cells were centrifuged at 3900× *g* for 5 min; pelleted bacteria were rinsed in 10 mM MgSO_4_ and diluted in Knop solution. The final PGPB concentration was monitored by counting the bacterial colonies grown on LB–agar medium and was 3 × 10^8^ CFU/mL. Wheat plants were inoculated with the bacterial strain *Bacillus* sp. *V2026* twice: at the time of planting and at the tillering stage. Bacterial cell suspension at a rate of 1 mL (3 × 10^8^ CFU/mL) per seedling was applied to the soil surface around the roots of each plant. In total, 5 mL of bacterial suspension in a concentration of 3 × 10^8^ CFU/mL was added to each pot.

Ontogenetic phases of spring soft wheat were observed using the conventional Eucarpia scale. Conducting vegetative experiments under controlled conditions allows more accurate determination of the time of onset of developmental phases [121,122]. The dates of the phases of development were observed individually for each plant. Tillering was noted on the day when the second shoot emerged from the main shoot. Stem elongation was recorded when the first node rose to a height of about 1 cm. Heading was recorded on the day when the ear fully emerged from the flag leaf. Days before the beginning of maturing were recorded as the number of days from the date of sowing to the date of yellowing of the upper internode of the main stem. The plants were harvested in the phase of full ripeness. In this study on wheat yield structure, data were analyzed on productive tilling capacity, plant height, spike length, number of spikelets in a spike, spike weight, and number and weight of grains per spike and per plant. After drying the samples at 70 °C for 48 h, the dry weight of the plant was determined. We calculated the weight of thousand grains, straw yield per plant (the difference between biological yield and grain yield), harvest index (HI, the ratio of grain yield to aboveground biomass yield expressed as a percentage), and the ratio of unproductive to productive spike weight (ratio chaff to grain).

#### 4.5.2. Hydroponic Experiment

Hydroponic experiments were performed in Knop solution (containing CaNO_3_ 1 g, KH_2_PO_4_ 0.25 g, MgSO_4_ 0.25 g, KCl 0.125 g, and FeCl_3_ 0.0125 g per 1 L) to study the effect of *Bacillus* sp. *V2026* on the growth of wheat plants and their biochemical and hormonal status. The experimental design included two variants: control—wheat plants without treatment grown on Knop medium; treatment—wheat plants grown in Knop solution and treated with *Bacillus* sp. *V2026* at a concentration of 3 × 10^5^ cells per·mL. The experiments were performed in three independent replications, and from 35 to 45 plants per variant were used in each replication.

For the experiment, undamaged and calibrated seeds were selected, surface-sterilized in 70% ethanol for 2 min, and washed with sterile water. The seeds were then soaked in 2% sodium hypochlorite solution for 20 min and washed five times with sterile water. After that, the seeds were placed in Petri dishes and germinated in a thermostat at 26 °C. After 48 h, the germinated seeds were placed between two layers of hydrophilic tissue at a distance of 2 cm from each other; then, the tissue was rolled up and placed in 300 mL vessels with Knop nutrient solution. For the hydroponic experiments, bacteria were diluted to a concentration of 3 × 10^5^ cells per 1 mL of Knop’s solution. On the 14th day after the beginning of the experiment and on the 10th day after adding the bacterium, the length and the biomass of plant shoots and roots were measured, and samples were taken to determine the content of photosynthetic pigments and hormone levels.

### 4.6. Analysis of Plant Phytohormones

To determine the phytohormones, 10 g of leaves were homogenized with 80% methanol at 4 °C and evaporated at a rotary evaporator under vacuum. The remaining aqueous phase was divided into two parts. One half was acidified with 10% solution of muriatic acid to pH 2.5–3 and extracted three times in a separating funnel with 30 mL diethyl ether for determination of IAA, tZ, and GA. The second part was diluted with 10% potassium hydroxide to pH 8.0 and extracted three times in a separating funnel with n-butanol (30 mL each), after which the extract was purified with Dowex 50W*8 ion-exchange resin. The final extracts were evaporated to a dry residue and dissolved in 2 mL of mobile phase A. Concentrations of IAA, tZ, and GA in the extract were determined by high-performance liquid chromatography with mass selective detection (VARIAN 212 LC liquid chromatograph with mass selective detector [Varian 500 MS system]). IAA was detected using an ESI- (electrospray) at 174 *m*/*z* ion. tZ and GA were detected using ESI+ (electrospray) at 220 *m*/*z* and 345 *m*/*z* ions, respectively. Chromatography was performed in gradient mode (phase A, methanol + 0.1% formic acid; phase B, deionized water +0.1% formic acid). Cosmosil C18 4.6 ID × 150 mm column was used in the chromatographic system. The chromatograph was calibrated using Sigma-Aldrich internal standards for pure hormones. The hormones were identified in MS/MS mode.

### 4.7. Chlorophyll and Carotenoids Analysis

Photosynthetic pigments chlorophyll a (Chl a), chlorophyll b (Chl b), and carotenoids (Car) were analyzed in acetone extract using the spectrophotometric method [123]. A total of 0.2 g of leaves were ground in a porcelain mortar with a small amount of acetone and sand in the presence of calcium carbonate. The ground mass was transferred to a centrifuge tube and centrifuged at 4000 rpm^−1^. The supernatant was transferred into a 50 mL volumetric flask and made up to the mark with acetone. Optical density was measured on a spectrophotometer PE-3000UF at 662, 644, and 440.5 nm wavelengths.

### 4.8. Lipid Peroxidation

The level of lipid peroxidation (LPO) was assessed based on the content of malonic dialdehyde (MDA), which is a product of LPO [124]. Plant material (0.3 g of raw leaves) was homogenized in 1 mL of reaction medium consisting of thiobarbituric acid and trichloroacetic acid. The total volume of the homogenate was 4 mL. One sample of reaction medium (4 mL) contained 0.4 g of trichloroacetic acid (10%) and 1.0 mg of thiobarbituric acid (0.25%). The homogenate was placed in a water bath at 95–100 °C for 30 min, after which the samples were cooled and centrifuged for 10 min at 10,000 g. Absorbance was measured at 532 nm and 600 nm. The TBA reactive product concentration was calculated using an extinction coefficient of 155 mM^−1^ cm^−1^.

### 4.9. Analysis of Protein and Macronutrient/Micronutrient Content in Wheat Grain

Wheat grain was dried in an oven to constant weight at a temperature of 105 °C, crushed in a mill, and sifted through a sieve with a mesh diameter of 1 mm. The prepared sample was used to determine the concentration of trace elements Fe, Mg, Zn, and Mn. For analysis, a sample weighing 1 g was transferred into precalcined crucibles, and dry ashing was carried out in a muffle furnace at a temperature of 520 °C for 5 h until complete ashing. After the crucibles cooled down, an 18% HCl solution was added to them, dissolving the ash; the contents of the crucibles were transferred with deionized water into a 100 mL volumetric flask. The resulting solution was filtered through an ash-free blue ribbon filter. The measurements were carried out on a Varian AA240FS atomic absorption spectrophotometer with flame atomization. The device was calibrated using standard solutions of elements with a given concentration. Trace elements were measured at the most sensitive wavelengths of Fe (248.7 nm), Mg (324.6 nm), Zn (213.7 nm), and Mn (279.5 nm) using hollow cathode lamps.

### 4.10. Statistical Analysis

For the statistical analysis, we used two-factor analysis of variance (ANOVA) and Duncan’s multiple range test to determine the significance of differences between the mean values. The number of repeats for each characteristic is shown in the tables and figures. The mean ± SE values presented in the tables and the figures were calculated using MS Excel.

## 5. Conclusions

PGPB *Bacillus* sp. *V2026* producing indole-3-acetic acid and gibberellin influenced the development dynamics and productivity of early-maturing cultivars S64 and LR and ultra-early-maturing lines AFI91 and AFI177 of wheat, as well as their physiological and biochemical responses and endogenous hormone levels at the early stages of ontogenesis. Inoculation with the bacterium significantly shortened the time to reach each growth stage in early-maturing genotypes, with cycle reduction being greater from seedlings to stem elongation. Inoculation of plants with *Bacillus* sp. *V2026* significantly affected the content of endogenous hormones IAA, GA, and tZ in roots and shoots of early-maturing wheat genotypes. The stimulating effect of *Bacillus* sp. *V2026* on the cultivars S64 and LR was mostly expressed as an increase in the number of grains, while the effect on the plants of lines AFI91 and AFI177 was mainly expressed as an increase in grain weight. The contents of macro- and microelements and protein in the grain of AFI91 and AFI177 were maximal compared with other genotypes. The bacterium *Bacillus* sp. *V2026* could be used to increase the yield and the grain quality of early-maturing genotypes of spring soft wheat. However, further studies are necessary to select the most effective association for growing high-yielding early-maturing wheat plants, since there are the differences in the response of early-maturing wheat genotypes to inoculation with PGPB *Bacillus* sp. *V2026*.

## Figures and Tables

**Table 1:** Effect of inoculation with *Bacillus* sp. *V2026* on yield attributes of early-maturing spring wheat.

Treatment	Main Stem				Plant						1000-Grain Weight, (g)
	Spike Length, (cm)	Number of Spikelets/Spike, (Number)	Number of Grains/Spike, (Number)	Grain Weight/Spike (g)	Plant Height (cm)	Number of Productive Tiller, (Number)s	Number of Grains/Plant, (Number)	Grain Yield, (g)	Straw Yield, (g)	Dry Weight, (g)	
Sonora 64											
Control	7.56 ± 0.22 ^d^	15.08 ± 0.48 ^c^	26.7 ± 1.5 ^f^	0.93 ± 0.06 ^d^	57.2 ± 0.7 ^cd^	5.20 ± 0.35 ^cd^	113.6 ± 8.7 ^c^	3.14 ± 0.28 ^bc^	4.27 ± 0.40 ^c^	7.41 ± 0.67 ^c^	27.6 ± 1.3 ^c^
*Bacillus* sp. *V2026*	8.24 ± 0.17 ^c^	16.16 ± 0.38 ^b^	32.3 ± 1.4 ^c^	1.09 ± 0.08 ^ab^	58.8 ± 1.2 ^bc^	6.48 ± 0.47 ^b^	138.8 ± 13.1 ^b^	4.16 ± 0.47 ^a^	4.93 ± 0.46 ^b^	9.09 ± 0.91 ^b^	30.1 ± 1.9 ^b^
AFI91											
Control	6.42 ± 0.18 ^g^	13.48 ± 0.40 ^f^	27.8 ± 0.9 ^ef^	0.94 ± 0.04 ^d^	56.2 ± 1.1 ^d^	5.08 ± 0.27 ^d^	106.9 ± 6.9 ^cd^	3.26 ± 0.22 ^bc^	3.62 ± 0.23 ^d^	6.88 ± 0.44 ^c^	30.8 ± 1.7 ^b^
*Bacillus* sp. *V2026*	6.64 ± 0.23 ^fg^	14.24 ± 0.32 ^de^	30.3 ± 1.1 ^d^	1.15 ± 0.04 ^a^	59.5 ± 1.2 ^b^	5.84 ± 0.33 ^c^	133.4 ± 9.7 ^b^	4.41 ± 0.37 ^a^	4.60 ± 0.38 ^bc^	9.01 ± 0.74 ^b^	33.0 ± 1.2 ^a^
AFI177											
Control	6.82 ± 0.17 ^ef^	13.76 ± 0.28 ^ef^	26.4 ± 1.1 ^f^	0.84 ± 0.04 ^e^	46.2 ± 0.5 ^e^	4.36 ± 0.29 ^e^	92.2 ± 5.5 ^d^	2.48 ± 0.14 ^d^	2.72 ± 0.17 ^f^	5.21 ± 0.29 ^d^	27.1 ± 1.1 ^c^
*Bacillus* sp. *V2026*	7.06 ± 0.14 ^e^	14.64 ± 0.22 ^cd^	28.8 ± 1.3 ^de^	1.06 ± 0.06 ^bc^	47.3 ± 0.5 ^e^	5.28 ± 0.49 ^cd^	107.6 ± 9.0 ^c^	3.38 ± 0.25 ^b^	3.44 ± 0.25 ^e^	6.82 ± 0.49 ^c^	31.7 ± 0.9 ^ab^
Leningradskaya rannyaya											
Control	8.92 ± 0.23 ^b^	17.76 ± 0.42 ^b^	34.2 ± 1.3 ^bc^	0.89 ± 0.03 ^de^	86.5 ± 1.9 ^a^	5.72 ± 0.48 ^cd^	138.7 ± 10.7 ^b^	2.83 ± 0.25 ^cd^	4,69 ± 0.37 ^bc^	7.52 ± 0.61 ^c^	20.4 ± 0.9 ^d^
*Bacillus* sp. *V2026*	9.48 ± 0.20 ^a^	19.08 ± 0.35 ^a^	39.9 ± 1.6 ^a^	1.01 ± 0.04 ^c^	85.6 ± 2.2 ^a^	7.28 ± 0.57 ^a^	219.1 ± 13.9 ^a^	4.59 ± 0.25 ^a^	5.60 ± 0.39 ^a^	10.19 ± 0.62 ^a^	21.1 ± 0.4 ^d^

Control, noninoculated wheat plants; *Bacillus* sp. *V2026*, wheat plants inoculated with *Bacillus* sb. *V2026*. The bars are means of two experiments with 50 biological replications per variant. Bars show ±SEM, and different letters ^(a–g)^ show a significant difference at the *p* ≤ 0.05 level, as determined by Duncan’s test.

**Table 2:** Effect of inoculation with *Bacillus* sp. *V2026* on macro- and micronutrient content of wheat grain.

Treatment	N, % DW	P, % DW	K, % DW	Mg, % DW	Fe, mg/kg^−^ DW	Mn, % DW	Zn, % DW
Sonora 64							
Control	3.11 ± 0.05 ^f^	0.61 ± 0.014 ^b^	0.61 ± 0.014 ^d^	0.242 ± 0.003 ^b^	43.7 ± 1.52 ^e^	47.1 ± 0.60 ^d^	49.8 ± 1.59 ^d^
*Bacillus* sp. *V2026*	3.46 ± 0.06 ^e^	0.63 ± 0.009 ^b^	0.75 ± 0.009 ^b^	0.224 ± 0.003 ^d^	44.6 ± 2.49 ^e^	50.5 ± 1.45 ^bc^	49.9 ± 1.73 ^d^
AFI91							
Control	3.09 ± 0.05 ^f^	0.56 ± 0.014 ^c^	0.56 ± 0.009 ^e^	0.223 ± 0.003 ^d^	55.0 ± 2.27 ^d^	52.3 ± 1.40 ^ab^	54.7 ± 1.17 ^c^
*Bacillus* sp. *V2026*	3.56 ± 0.06 ^d^	0.62 ± 0.009 ^b^	0.68 ± 0.014 ^c^	0.232 ± 0.003 ^c^	62.8 ± 1.02 ^b^	53.8 ± 1.57 ^a^	58.4 ± 0.83 ^b^
AFI177							
Control	3.44 ± 0.04 ^e^	0.62 ± 0.009 ^b^	0.76 ± 0.014 ^b^	0.243 ± 0.004 ^b^	58.3 ± 1.01 ^c^	49.0 ± 1.78 ^cd^	53.4 ± 0.77 ^c^
*Bacillus* sp. *V2026*	4.13 ± 0.04 ^a^	0.68 ± 0.014 ^a^	0.88 ± 0.016 ^a^	0.254 ± 0.005 ^a^	69.3 ± 1.76 ^a^	53.4 ± 1.05 ^a^	55.4 ± 1.65 ^c^
Leningradskaya rannyaya							
Control	3.70 ± 0.04 ^c^	0.69 ± 0.005 ^a^	0.58 ± 0.014 ^e^	0.252 ± 0.002 ^a^	43.2 ± 1.02 ^e^	50.8 ± 0.93 ^bc^	62.8 ± 1.02 ^a^
*Bacillus* sp. *V2026*.	3.93 ± 0.05 ^b^	0.68 ± 0.009 ^a^	0.62 ± 0.014 ^d^	0.231 ± 0.003 ^c^	41.8 ± 1.30 ^e^	52.5 ± 1.22 ^ab^	62.0 ± 0.65 ^a^

Control, noninoculated wheat plants; *Bacillus* sp. *V2026*, wheat plants inoculated with *Bacillus* sp. *V2026*; DW, dry weight. Values in columns followed by different letters ^(a–^^f)^ are significantly different at *p* ≤ 0.05 (Duncan’s test).

**Table 3:** Effect of inoculation with *Bacillus* sp. *2026* on the duration of developmental phases of early-maturing spring wheat.

Treatment	Seedling–Tillering, Days	Tillering–Stem Elongation, Days	Seedling– Stem Elongation, Days	Stem Elongation– Heading, Days	Seedling– Heading, Days	Heading– Maturating, Days	Seedling– Maturating, Days
Sonora 64							
Control	11.3 ± 0.24 ^b^	5.8 ± 0.23 ^bc^	17.0 ± 0.23 ^b^	22.9 ± 0.28 ^b^	40.0 ± 0.23 ^b^	33.3 ± 0.43 ^c^	73.2 ± 0.50 ^a^
*Bacillus* sp. *V2026*	10.6 ± 0.22 ^e^	5.1 ± 0.35 ^d^	15.6 ± 0.31 ^c^	22.8 ± 0.48 ^b^	38.5 ± 0.37 ^c^	33.2 ± 0.46 ^c^	71.7 ± 0.49 ^b^
AFI91							
Control	11.7 ± 0.28 ^a^	2.9 ± 0.27 ^f^	14.6 ± 0.27 ^d^	16.6 ± 0.32 ^e^	31.2 ± 0.35 ^f^	37.1 ± 0.51 ^a^	68.3 ± 0.49 ^e^
*Bacillus* sp. *V2026*	11.1 ± 0.20 ^bc^	2.3 ± 0.30 ^g^	13.4 ± 0.29 ^e^	16.4 ± 0.25 ^e^	29.8 ± 0.24 ^g^	36.5 ± 0.33 ^a^	66.3 ± 0.29 ^f^
AFI177							
Control	10.8 ± 0.23 ^de^	6.2 ± 0.29 ^a^	16.9 ± 0.27 ^b^	18.6 ± 0.29 ^c^	35.5 ± 0.20 ^d^	35.2 ± 0.41 ^b^	70.7 ± 0.48 ^c^
*Bacillus* sp. *V2026*	10.2 ± 0.16 ^f^	5.4 ± 0.35 ^cd^	15.6 ± 0.27 ^c^	17.5 ± 0.45 ^d^	33.1 ± 0.37 ^e^	35.7 ± 0.56 ^b^	68.8 ± 0.75 ^e^
Leningradskaya rannyaya							
Control	11.9 ± 0.13 ^a^	5.9 ± 0.31 ^b^	17.8 ± 0.31 ^a^	24.4 ± 0.46 ^a^	42.2 ± 0.44 ^a^	30.3 ± 0.64 ^d^	72.4 ± 0.78 ^ab^
*Bacillus* sp. *V2026*	10.9 ± 0.15 ^cd^	4.0 ± 0.28 ^e^	14.9 ± 0.26 ^d^	24.2 ± 0.42 ^a^	39.1 ± 0.40 ^c^	30.6 ± 0.46 ^d^	69.7 ± 0.57 ^d^

Control, noninoculated wheat plants; *Bacillus* sp. *V2026*, wheat plants inoculated with *Bacillus* sp. *V2026*. The bars are means of two experiments with 50 biological replications per variant. Bars show ± SEM, and different letters ^(a–f)^ show a significant difference at the *p* ≤ 0.05 level, as determined by Duncan’s multiple test.

**Table 4:** Effect of *Bacillus* sp. *V2026* on wheat roots and shoots of early-maturing wheat in hydroponic conditions.

Treatment	Length, mm		Fresh Weight, mg		Root/Shoot (mm/mm)	Root/Shoot (mg/mg)
	Root	Shoot	Root	Shoot		
Sonora 64						
Control	169.7 ± 5.30 ^c^	279.4 ± 8.39 ^c^	25.6 ± 1.15 ^b^	36.7 ± 1.22 ^b^	0.61 ^d^	0.69 ^d^
*Bacillus* sp. *V2026*	189.1 ± 7.61 ^b^	295.9 ± 7.71 ^b^	28.4 ± 1.40 ^a^	39.9 ± 1.75 ^a^	0.64 ^c^	0.71 ^c^
AFI91						
Control	134.7 ± 4.46 ^d^	281.6 ± 9.40 ^c^	19.6 ± 1.86 ^e^	31.4 ± 1.45 ^cd^	0.48 ^f^	0.62 ^f^
*Bacillus* sp. *V2026*	160.3 ± 6.15 ^c^	283.8 ± 11.21 ^c^	22.1 ± 1.65 ^cd^	33.3 ± 1.82 ^c^	0.56 ^e^	0.66 ^e^
AFI177						
Control	166.3 ± 7.63 ^c^	181.9 ± 3.23 ^d^	20.4 ± 1.07 ^de^	24.9 ± 0.96 ^e^	0.91 ^b^	0.82 ^b^
*Bacillus* sp. *V2026*	200.3 ± 5.23 ^a^	192.2 ± 6.54 ^d^	25.1 ± 1.01 ^b^	29.6 ± 1.58 ^d^	1.04 ^a^	0.85 ^a^
Leningradskaya rannyaya						
Control	160.0 ± 5,94 ^c^	283.7 ± 7.5 ^c^	19.4 ± 1.01 ^e^	29.1 ± 1.63 ^d^	0.56 ^e^	0.67 ^e^
*Bacillus* sp. *V2026*	193.1 ± 7.08 ^ab^	313.1 ± 5.54 ^a^	23.7 ± 1.40 ^bc^	33.0 ± 1.60 ^c^	0.62 ^d^	0.72 ^c^

Control, noninoculated wheat plants; *Bacillus* sp. *V2026*, wheat plants inoculated with *Bacillus* sp. *V2026*. Values in columns followed by different letters ^(a–f)^ are significantly different at *p* ≤ 0.05 (Duncan’s test).

## Data Availability

The data presented in this study are available upon request from the corresponding author.

## Supplementary Materials

The following supporting information can be downloaded at: https://www.mdpi.com/article/10.3390/plants11141817/s1. Table S1: morphological, physiological, and biochemical characteristics of the studied strain, Table S2: analysis of variance (ANOVA) for genotype and *Bacillus* sp. *V2026* effect on various traits of four spring wheat genotypes, Table S3, primers used in the study, Figure S1: identification of dominant (288 b.p.) and recessive (414 b.p.) alleles of *Ppd-D1* gene (a), dominant (715+624 b.p.) and recessive (484 b.p.) alleles of *Vrn-A1* gene (b), dominant (1124 b.p.) allele of *Vrn-B1* gene (c), and dominant (1671 b.p.) allele of *Vrn-D1* gene (d) in wheat varieties by PCR with allele-specific markers. Wheat varieties: 1- AFI-177, 2- AFI-91, 3- Leningradskaya rannayay, 4- Sonora-64, M-DNA ladder; Figure S2, effect of inoculation with *Bacillus* sp. *V2026* on wheat seedlings of early-maturing genotypes.
