# Supplementary material for: Plant Development of Early-Maturing Spring Wheat (Triticum aestivum L.) under Inoculation with Bacillus sp. V2026
Source: Plants (Basel). 2022 Jul 10;11(14):1817. doi: 10.3390/plants11141817 (PMC9317556; doi:10.3390/plants11141817)
Supplement: Supplementary file 1 [file plants-11-01817-s001.zip › plants-1786999-supplementary.pdf]

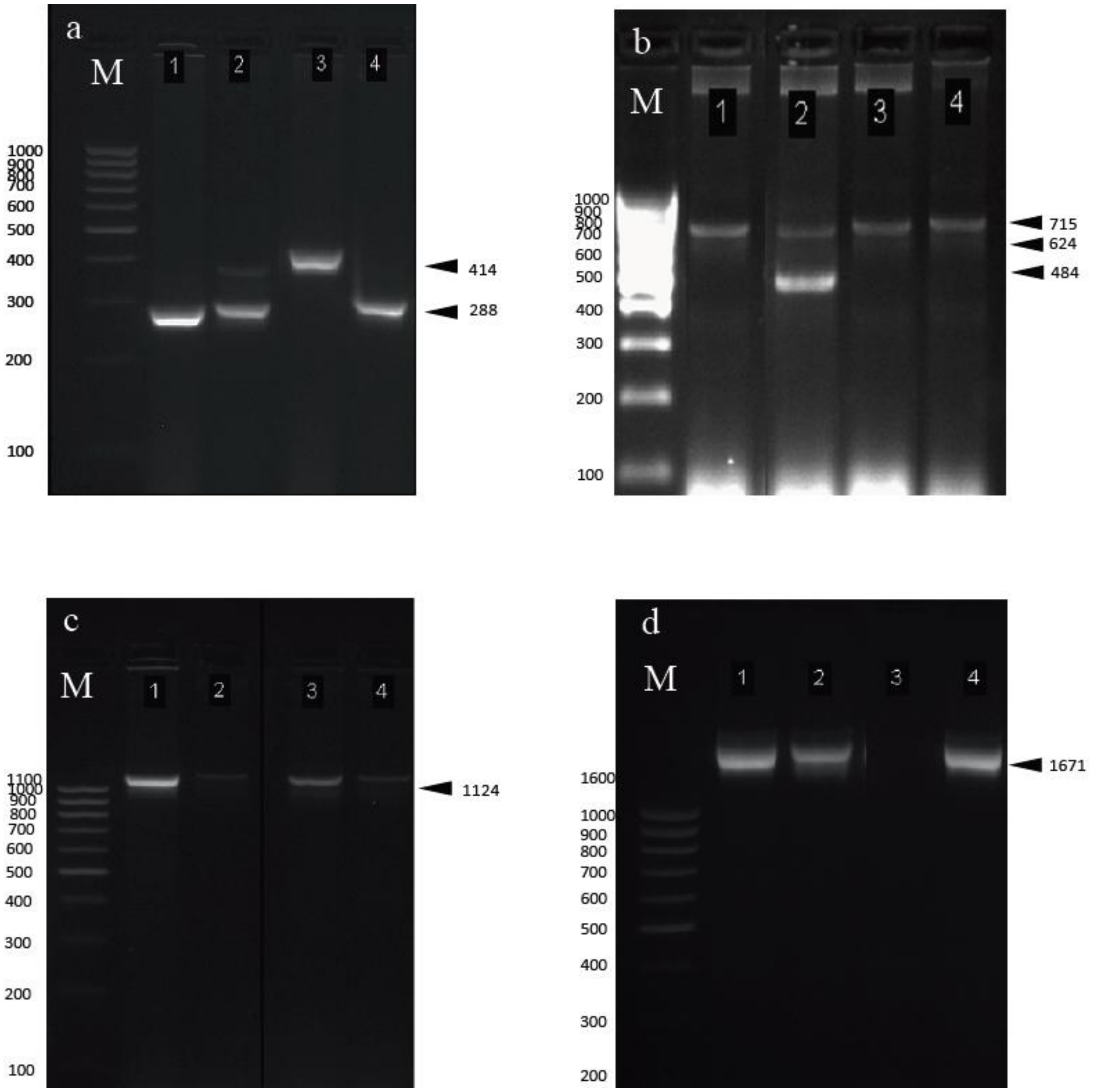

Figure S1. Identification of dominant (288 b.p.) and recessive (414 b.p.) alleles of *Ppd-D1*gene(**a**), dominant (715+624 b.p.) and recessive (484 b.p.) alleles of *Vrn-A1* gene(**b**), dominant (1124 b.p.) allele of *Vrn-B1* gene(**c**), and dominant (1671 b.p.) allele of *Vrn-D1* gene(**d**), in wheat varieties by PCR with allele-specific markers. Wheat varieties: 1- AFI-177, 2- AFI-91, 3- Leningradskaya rannya, 4- Sonora-64; M-DNA ladder.

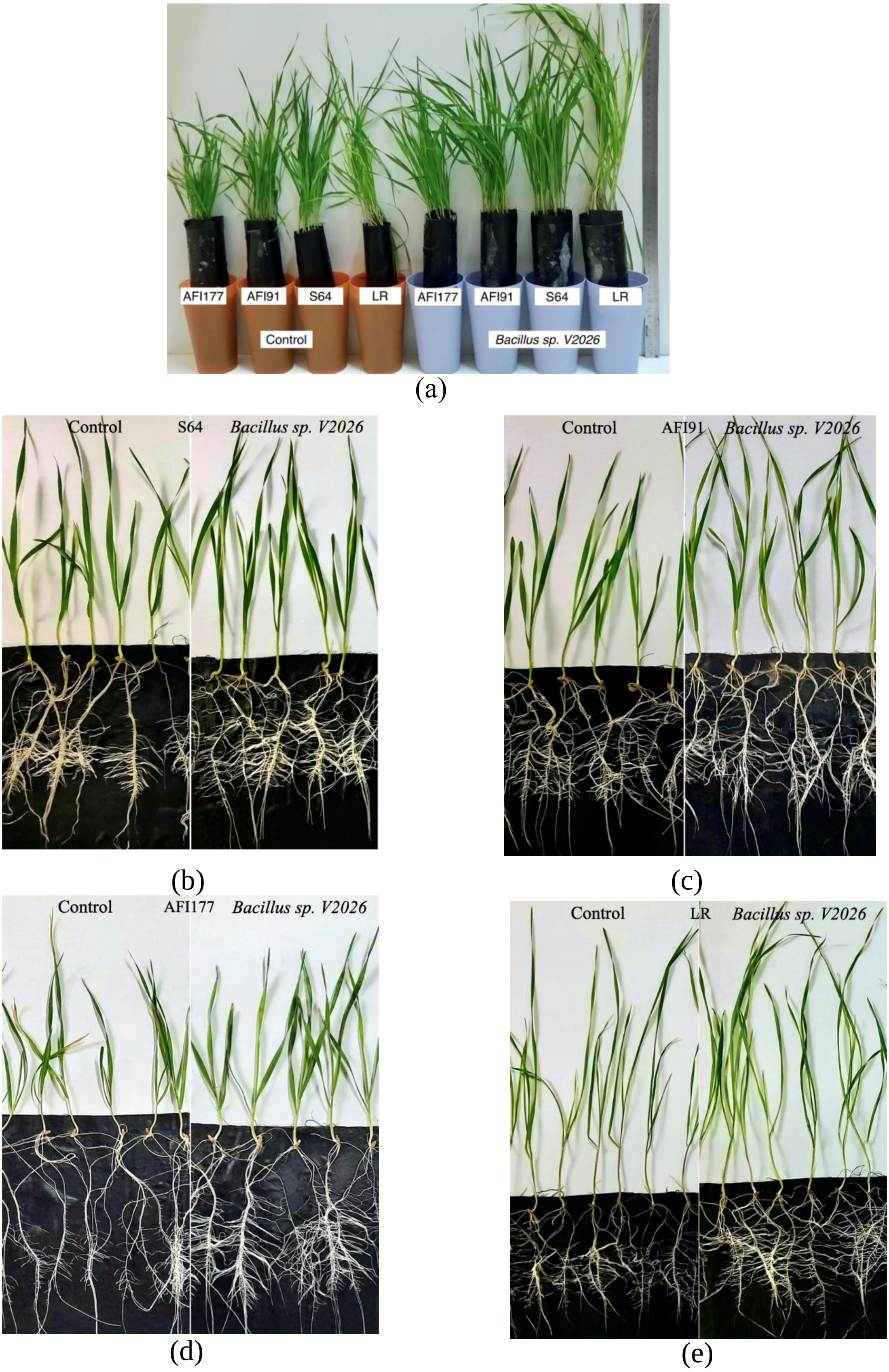

Figure S2. Effect of inoculation with *Bacillussp. V2026* on wheat seedlings of early-maturing genotypes (**a**): 14-day old wheat seedlings in hydroponic experiments; (**b**): plants of cv.S64 without treatment (control) and inoculated with *Bacillussp. V2026;* (**c**): ultra-early-maturingline AFI91 without treatment (control) and inoculated with *Bacillussp. V2026* plants*;* (**d**):ultra-early-maturingline AFI177 without treatment(control) and inoculated with *Bacillussp. V2026* plants*;* (**e**): plants of cv.LR without treatment (control) and inoculated with *Bacillussp. V2026.*

Table S1. Morphological, physiological and biochemical characteristics of the studied strain

**Characteristics**	***Bacillus sp. V2026***
Gram staining	+
Cell shape	Rod
Spore-forming	+
Morphology of colonies	Round, white, smooth
Motile	+
Oxidase	+
Catalase	-
Indole	-
H_2S	-
Growth at 5^0C	+
Growth at 40^0C	+
Growth on 6% NaCl	+
Voges-Proskauer	-
**Utilization of**	
Glucose	+
Sucrose	+
xylose	+
arabinose	+
maltose	+
sorbitol	+
mannitol	+

Table S2. Analysis of variance (ANOVA) for genotype and *Bacillus sp.V2026* effect on various traits four spring wheat genotypes

Trait	Source of Variation	df	MS	F-value	P-value
Productivity Wheat yield traits					
Plant height	Genotype	3	14090.0	1251.2	0.001
	Treatment	1	81.9	7.27	0.01
	Genotype x Treatment	3	36.9	3.28	0.05
Spike length	Genotype	3	70.39	275.81	0.001
	Treatment	1	9.03	35.39	0.001
	Genotype x Treatment	3	0.66	2.60	ns
Number of prodactive tillers	Genotype	3	24.73	20.79	0.001
	Treatment	1	63.85	53.69	0.001
	Genotype x Treatment	3	1.62	1.361	ns
Number of spikelets/ spike	Genotype	3	215.3	239.33	0.001
	Treatment	1	51.01	56.70	0.001
	Genotype x Treatment	3	0.75	0.84	ns
Number of grains/ spike	Genotype	3	894.2	78.19	0.001
	Treatment	1	816.1	71.36	0.001
	Genotype x Treatment	3	43.8	3.83	0.05
Grain weight/ spike	Genotype	3	0.108	6.20	0.001
	Treatment	1	1.621	93.36	0.001
	Genotype x Treatment	3	0.023	1.30	ns
Number of grains/ plant	Genotype	3	56698	82.49	0.001
	Treatment	1	67896	98.79	0.001
	Genotype x Treatment	3	10830	15.76	0.001
Grain yield	Genotype	3	8.39	14.08	0.001
	Treatment	1	72.92	122.35	0.001
	Genotype x Treatment	3	1.81	3.04	0.05
Harvest index	Genotype	3	0.060	94.28	0.001
	Treatment	1	0.062	98.58	0.001
	Genotype x Treatment	3	0.010	15.82	0.001
1000 grain weight	Genotype	3	1169.4	103.73	0.001
	Treatment	1	303.5	26.92	0.001
	Genotype x Treatment	3	34.0	3.02	0.05
Straw yield	Genotype	3	38.30	47.06	0.001
	Treatment	1	33.33	40.95	0.001
	Genotype x Treatment	3	0.30	0.37	ns
Chaff to grain ratio	Genotype	3	0.008	7.41	0.001
	Treatment	1	0.193	185.17	0.001
	Genotype x Treatment	3	0.006	5.97	0.001
Onthogenesis					
Seedling-tillering	Genotype	3	9.93	33.86	0.001
	Treatment	1	25.92	88.36	0.001
	Genotype x Treatment	3	0.41	1.41	ns
Tillering-stem elongation	Genotype	3	109.50	177.69	0.001
	Treatment	1	60.50	98.17	0.001
	Genotype x Treatment	3	4.70	7.63	0.001
Seedling-stem elongation	Genotype	3	69.71	129.90	0.001
	Treatment	1	165.62	308.61	0.001
	Genotype x Treatment	3	7.13	13.28	0.001

Stem elongation-heading	Genotype	3	715.36	725.03	0.001
	Treatment	1	3.38	3.43	ns
	Genotype x Treatment	3	1.41	1.43	ns
Seedling-heading	Genotype	3	1076.4	1400.9	0.001
	Treatment	1	214.2	278.8	0.001
	Genotype x Treatment	3	8.2	10.7	0.001
Heading-maturating	Genotype	3	385.6	205.24	0.001
	Treatment	1	ns	0.01	ns
	Genotype x Treatment	3	ns	1.49	ns
Seedling-maturating	Genotype	3	242.5	104.1	0.001
	Treatment	1	210.1	90.2	0.001
	Genotype x Treatment	3	3.3	1.4	ns
Protein and macronutrient/micronutrient content in grain					
N	Genotype	3	0.5027	188.8	0.001
	Treatment	1	1.1008	413.5	0.001
	Genotype x Treatment	3	0.0597	22.4	0.001
P	Genotype	3	0.009126	64.42	0.001
	Treatment	1	0.005704	40.26	0.001
	Genotype x Treatment	3	0.001471	10.38	0.001
K	Genotype	3	0.06110	293.30	0.001
	Treatment	1	0.06510	312.50	0.001
	Genotype x Treatment	3	0.00265	12.71	0.001
Mg	Genotype	3	0.000555	41.3	0.001
	Treatment	1	0.000081	6.0	0.05
	Genotype x Treatment	3	0.000377	28.1	0.001
Fe	Genotype	3	677.08	223.37	0.001
	Treatment	1	124.22	40.98	0.001
	Genotype x Treatment	3	50.66	16.71	0.001
Mn	Genotype	3	18.33	9.28	0.001
	Treatment	1	45.65	23.11	0.001
	Genotype x Treatment	3	2.88	1.46	ns
Zn	Genotype	3	162.92	83.49	0.001
	Treatment	1	9.13	4.68	0.05
	Genotype x Treatment	3	5.97	3.06	ns
Protein	Genotype	3	19.64	188.8	0.001
	Treatment	1	43.00	413.5	0.001
	Genotype x Treatment	3	2.33	22.4	0.001
Hormones					
IAA in roots	Genotype	3	618.73	52.00	0.001
	Treatment	1	532.04	44.72	0.001
	Genotype x Treatment	3	91.90	7.72	0.01
IAA in shoots	Genotype	3	115.42	25.31	0.001
	Treatment	1	1159.26	254.15	0.001
	Genotype x Treatment	3	73.97	16.22	0.001
GA in roots	Genotype	3	199.79	100.43	0.001
	Treatment	1	2398.00	1205.42	0.001
	Genotype x Treatment	3	173.40	87.16	0.001
GA in shoots	Genotype	3	84.43	136.50	0.001
	Treatment	1	133.58	215.96	0.001

	Genotype x Treatment	3	9.78	15.81	0.001
tZ in roots	Genotype	3	97.52	187.623	0.001
	Treatment	1	98.29	189.11	0.001
	Genotype x Treatment	3	199.95	384.69	0.001
tZ in shoots	Genotype	3	10.959	144.77	0.001
	Treatment	1	0.788	10.412	0.01
	Genotype x Treatment	3	10.344	136.64	0.001

Table S3. Primers used in the study

**Plants primers**			
Allel	Allele-specific primers (5’-3’)	Fragment length, bp	Reference
Ppd-D1a<br><br>*Ppd-D1b*	***Ppd-D1A*F**<br>ACGCCTCCCACTACACTG<br>***Ppd-D1*/R1**<br>GTTGGTTCAAACAGAGAGC<br>***Ppd-D1*/R2**<br>CACTGGTGGTAGCTGAGATT	288<br><br>414	Beales et al.,2007
Vrn-A1a<br><br><br><br>*vrn-A1*	**VRN1AF**<br>GAAAGGAAAAATTCTGCTCG<br><br>**VRN1-1R**<br>TGCACCTTCCCCCGCCCCAT	715 + 624<br><br>484	Yan et al., 2004
Vrn-B1a	**Intr1**<br>ATCATCTTCTCCACCAAGGG<br><br>**Intr1/B/R3**<br>CTCATGCCAAAAATTGAAGATGA	1124	Fu et al., 2005
Vrn-D1	**Intr1/D/F**<br>GTTGTCTGCCTCATCAAATCC<br><br>**Intr1/D/R3**<br>GGTCACTGGTGGTCTGTGC	1671	Fu et al., 2005

**Bacterial primers**		
Genome fragment	Primers (5’-3’)	Reference
16S rRNA	**27f**<br>AGAGTTTGATCMTGGCTCAG<br><br>**1525r**<br>AAGGAGGTGWTCCARCC	Lane, 1991
ITS	**FGPL132-38**<br>CCGGGTTTCCCCATTCGG<br><br>**FGPS1490-72**<br>TGCGGCTGGATCACCTCCTT	Normand et al., 1992
